# Getting to full disclosure: HCV testing and status disclosure behaviors among PWID and their injecting partners

**DOI:** 10.1186/s12889-025-22781-6

**Published:** 2025-05-08

**Authors:** Maia Scarpetta, Rachel Kanner, Neia Prata Menezes, Claire C. McDonell, Julie Bruneau, Kimberly Page, Meghan D. Morris

**Affiliations:** 1 Department of Epidemiology and Biostatistics, University of California, San Francisco, Box 1224, San Francisco, CA 550 16thStreet94158 USA; 2 Department of Medicine, University of California San Francisco, San Francisco, USA; 3 Center for AIDS Prevention Sciences (CAPS), University of California, San Francisco, San Francisco, USA; 4 Department of Family Medicine and Emergency Medicine, Université de Montréal, Montréal, Canada; 5 Department of Internal Medicine, University of New Mexico Health Sciences Center, Albuquerque, NM USA

**Keywords:** Hepatitis C virus, People who inject drugs, HCV testing, HCV disclosure, Injecting partnerships

## Abstract

**Background:**

People who inject drugs (PWID) face a substantial risk of hepatitis C virus (HCV) infection, often in the context of multiple injecting partnerships. The disclosure of HCV status to injecting partners holds significant implications for prevention and care among PWID.

**Methods:**

We used cross-sectional dyadic survey data (collected from both members of injecting partnerships) to estimate the prevalence of HCV-status disclosure between PWID and their injecting partners, overall and by partnership HCV infection status.

**Results:**

Across the two study sites (San Francisco and Montreal), 91% of participants self-reported receiving an HCV test, resulting in 162 individuals and 131 partnerships. A majority (57%) self-reported being HCV positive. HCV status disclosure was prevalent overall (79%) and was most common (41%) with partnerships where both partners’ status was positive (+ / +) but less common (17%) when one partner was positive ( ±) and when neither partner was positive (-/-) (32%); no disclosure was more common when both partners were negative (-/-) (50%).

**Conclusions:**

Overall, our study demonstrated a high prevalence of HCV testing and subsequent disclosure of HCV status within injecting partnerships. This presents an opportunity to leverage these relationships for treatment linkage and prevention messaging.

## Background

Despite significant advancements in the treatment of hepatitis C virus (HCV) over the last 30 years, chronic HCV infection continues to affect approximately 1% of the U.S. population, or between 2.1 to 4.7 million people [1]. Before the COVID-19 pandemic, HCV was the leading cause of death related to infectious diseases in the United States, with an average mortality rate of 4.13 deaths per 100,000 person-years between 2016 and 2017 [2]. Recent surveillance data show that the number of HCV cases in the U.S. has more than doubled from 2014 to 2020, and rates increased by 7% from 2020 to 2021 [3]. People who inject drugs (PWID) make up the largest share of new HCV infections, with HCV prevalence among PWID estimated at 70–90% [4–6]. PWID often live in marginalized conditions where structural barriers make it difficult to access healthcare services, such as HCV testing and counseling for results [7–9].

HCV is primarily transmitted through the sharing of drug-injecting equipment [10]. Among PWID, HCV infection often occurs within injecting partnerships—two or more people who regularly inject drugs together—where individuals may share needles, syringes, and other equipment [11]. A fundamental barrier to HCV status disclosure is the lack of awareness about HCV, compounded by the two-step diagnostic process [12]. The initial rapid fingerstick HCV antibody test is practical but necessitates a follow-up venipuncture HCV RNA test for infection diagnosis.^1^ Additionally, spontaneous clearance of HCV infection and the potential for reinfection complicate disclosure practices [4, 13]. Because a subset of individuals can spontaneously clear the infection without treatment and reinfection remains possible, regular testing is essential to ensure individuals are accurately reporting their current infection status [14, 15]. By examining self-reported disclosure patterns within injecting partnerships, this study aims to provide insights into the role of HCV status disclosure in prevention efforts and how perceived versus actual infection status may shape risk behaviors.

Interpersonal communication within the social networks of PWID has been identified as a critical factor for increasing HCV awareness and facilitating access to care, particularly when trust and frequent interaction are present in these relationships [16], but little research exists on HCV status disclosure between injecting partners or the underlying factors that lead to such disclosure. One study of rural PWID revealed low rates of HCV disclosure to injection partners, underscoring the need for interventions that encourage status sharing to prevent transmission within these high-risk dyads [17]. This information could help to refine counseling to be more inclusive of partnerships. HCV status disclosure between injecting partners permits each partner to make informed decisions about how they use drugs together and encourages partners to test early, often, and routinely [18–20]. Partnership disclosure is also crucial as injecting partners can be potential sources for support around managing HCV diagnosis and infection symptoms, accessing and adhering to HCV treatment [21, 22].

In this study, we examined HCV status disclosure within injecting partnerships, focusing on disclosure patterns based on the partnership’s HCV status. It focuses on self-reported HCV status disclosure, a key step in the diagnostic process that can potentially reduce stigma, promote HCV testing in others, and open more opportunities for treatment [23]. We used baseline data from a cross-sectional sample of PWID and their injecting partners to estimate the prevalence of HCV disclosure within partnerships, assessing whether one, both, or neither partner disclosed their status, overall and by infection status.

## Methods

### Study sample

This nested study used baseline data from the Partner Study, an epidemiological study of drug use and HCV among injecting partnerships (two people who inject drugs together) conducted between 2016 and 2019 in San Francisco, USA, and Montreal, Canada.

The Partner Study utilized infrastructure within two established cohort studies of PWID, the U-Find-Out (UFO) Study of young adult (≤ 30 years of age) PWID in San Francisco, USA, and the Hepatitis Cohort (HEPCO) Study of PWID in Montreal, Canada [19, 24]. Cohort participants were invited to participate in an eligibility screening assessment with their injecting partners for participation in the Partner Study. All Partner Study activities occurred at the parent study site in the city center and central to transportation and were conducted by the parent study staff. Study inclusion criteria required that partners had injected drugs at least three times together in the same physical space in the past month and pass a partnership validation assessment where injecting partners were separately asked about their own and their injecting partner’s demographic and injecting behaviors. Participants could be enrolled with up to three injecting partners at any one time. Partners were also consented separately to ensure the decision to participate was made independently.

Separately, both members of the injecting partnership completed research staff-administered behavioral surveys about their knowledge and disclosure of their own and their partner’s HCV and HIV status and underwent testing to detect HCV and HIV infection. HCV and HIV status for our substudy was based solely on participant’s self-reported status reflecting their perceived HCV status before study enrollment.

Participants received cash compensation for their time (30 USD, 20 CAD). All research protocols were reviewed and approved by the University of California, San Francisco Institutional Review Board, and the Centre Hospitalier de l’Universite de Montreal (CHUM) (Study number: 14–12999).

### Measures

We analyzed baseline behavioral survey data, excluding participants who reported never having a previous HCV test or had missing data for this question (34 participants).

HCV Status Disclosure: We created a three-level dyadic measure of HCV status disclosure within injecting partnerships by pairing responses to the question, "Have you told your partner your HCV status?" This measure includes Mutual HCV status disclosure, one-way HCV status disclosure, and no HCV status disclosure.

HCV Status: Among injecting partnership members where at least one member self-reported ever having an HCV test, we used their responses to "What was your most recent hepatitis C test result?" to classify their current HCV infection status as positive, negative, or unknown. Self-report data used in this analysis for two main reasons: 1) self-report reflects an individual’s perceived HCV status, regardless of their actual testing results; 2) HCV testing administered by the study occurred after the behavioral survey, therefore all study questions related to disclosure to partner were based on the individual’s perceived HCV status prior to being re-tested in the study. Additionally, self-reported HCV status is relevant in behavioral studies due to potential disparities between actual serological status and individuals' perception of HCV status [2, 3]. Participants were also asked about HCV treatment, though the type of treatment (e.g., direct-acting antivirals, DAAs) was not specified.

The same questions were posed regarding HIV testing and HIV status disclosure within partnerships. Individual demographic variables encompassed age, gender, race/ethnicity, sexual orientation, education, and country of origin. Partnership characteristics encompassed cohabitation (staying together in the same space for at least one night) and engagement in a sexual relationship (having had vaginal or anal sex with the partner in the past 30 days). Partnership composition measures were derived by aggregating responses from both partnership members regarding select socio-demographic factors (e.g., age, gender).

### Analyses

We employed standard descriptive statistics to examine HCV and HIV status disclosure, testing, and treatment characteristics, and individual and partnership traits. We utilized the Kruskal–Wallis test for nonparametric continuous variables and the Pearson chi-squared test for categorical variables, using Fisher’s exact test when any expected cell count was less than 5, for bivariate comparisons across study sites.

In this analysis, we solely conducted descriptive statistics since our research focused on characterizing this cross-sectional sample of PWID and determining the prevalence of their behaviors concerning HCV status disclosure. Regression analysis was omitted due to the small sample size and high prevalence of status disclosure. Based on the moderate (> 15%) amount of missing data in our analytic variables, we included missing values in the analysis and reported them in the tables for transparency and interpretability. Given that self-reported HCV status and disclosure behaviors were key measures, missing data could reflect meaningful differences in knowledge, awareness, or willingness to disclose among participants, rather than random omissions.

## Results

In the Partner Study, a total of 179 individuals representing 131 injecting partnerships participated: 79 individuals from the San Francisco site, representing 80 partnerships, and 83 individuals from the Montreal site, representing 51 partnerships. Among these participants, 162 individuals (91%) reported ever being tested for HCV. Participants could be enrolled in up to 3 partnerships, and 59 participants (36%) were enrolled in more than one partnership. Overall, the dataset had a modest amount of missing data, with the highest proportion of missing data was observed for self-reported HIV status (15%), while other key analytic variables had minimal missingness, as detailed in Tables 1 and 2.

**Table 1:** Individual-level characteristics (*N* = 162)

Characteristic	Overall, n (%) *N* = 162	San Francisco, n (%) *N* = 79	Montreal, n (%) *N* = 83	*p*-value^2^
Age^1^	29 (14.00)	26 (5.66)	37 (16.00)	< 0.001
Gender				
Male	118 (72.84)	58 (73.42)	60 (72.29)	0.227
Female	40 (24.69)	18 (22.78)	22 (26.51)	
Transgender, gender non-conforming, queer	1 (0.62)	0 (0.00)	1 (1.20)	
Missing	3 (1.85)	3 (3.80)	0 (0.00)	
Race/ethnicity^3^				
White	129 (79.63)	50 (63.29)	79 (95.18)	< 0.001
Non-white	32 (19.75)	28 (35.44)	4 (4.82)	
Missing	1 (0.62)	1 (1.27)	0 (0.00)	
Highest level of education				
Completed high school	116 (71.60)	64 (81.01)	52 (62.65)	0.015
Did not complete high school	46 (28.40)	15 (18.99)	31 (37.35)	
Missing	0 (0.00)	0 (0.00)	0 (0.00)	
HCV status (self-report)				
Positive	92 (56.79)	38 (48.10)	54 (65.06)	0.003
Negative	61 (37.65)	39 (49.37)	22 (26.51)	
Unknown/Missing	9 (5.55)	2 (2.53)	7 (8.43)	
HIV status (self-report)				
Positive	12 (7.41)	3 (3.80)	9 (10.84)	< 0.001
Negative	125 (77.16)	67 (84.81)	58 (69.88)	
Unknown/Missing	25 (15.43)	9 (11.39)	16 (19.28)	
Days injected in past month^1^	30 (10)	30 (6)	28 (15)	
Days injected in past month with partner^1^	20 (20)	20 (20)	20 (20)	
Times per day injected in past month^1^	3 (2)	3 (2)	3 (4)	
Number of people injected with in past month^1^	10 (22)	10 (22)	1 (3)	
Number of other injecting partners in past month^1^	3 (7)	4 (10)	1 (2)	

^1^Continuous measures are presented as median (IQR)

^2^Kruskal-Wallis test performed to generate *p*-values

^3^Race-ethnicity includes only white and non-white race categories due to the presence of very small or zero values for the complete race category breakdown

**Table 2:** Partnership-level characteristics (*N* = 131)

Characteristic	Overall, n (%) *N* = 131	San Francisco, n (%) *N* = 80	Montreal, n (%) *N* = 51	*p*-value
Partnership age composition				
Both under 30	62 (47.33)	57 (71.25)	5 (9.80)	< 0.001
One under 30	33 (25.19)	23 (28.75)	10 (19.61)	
Both 30 and over	36 (27.48)	0 (0.00)	36 (70.59)	
Missing	0 (0.00)	0 (0.00)	0 (0.00)	
Partnership gender composition				
Male/Male	73 (55.73)	42 (52.50)	31 (60.78)	0.172
Female/Female	23 (17.56)	17 (21.25)	6 (11.76)	
Male/Female	20 (15.27)	10 (12.50)	10 (19.61)	
Male/ Trans	1 (0.76)	0 (0.00)	1 (1.96)	
Missing	14 (10.69)	11 (13.75)	3 (5.88)	
Lived together in past month				
Yes	83 (63.36)	53 (66.25)	30 (58.82)	0.390
No	48 (36.64)	27 (33.75)	21 (41.18)	
Sex together in past month				
Yes	35 (26.72)	20 (25.00)	15 (29.41)	0.578
No	96 (73.28)	60 (75.00)	36 (70.59)	
Any equipment sharing within partnership in past month				
Yes	93 (70.99)	54 (67.50)	39 (76.47)	0.270
No	38 (29.01)	26 (32.50)	12 (23.47)	
Ever shared needle/syringe within partnership				
Yes	18 (13.74)	10 (12.50)	8 (15.69)	0.606
No	113 (86.26)	70 (87.50)	43 (84.31)	
Partnership HCV disclosure				
Both members disclosed	103 (78.63)	61 (76.25)	42 (82.35)	0.873
One member disclosed	16 (12.21)	11 (13.75)	5 (9.80)	
Neither members disclosed	6 (4.58)	4 (5.00)	2 (3.92)	
Missing	6 (4.58)	4 (5.00)	2 (3.92)	
Partnership HCV serostatus composition				
Concordant (-/-)	40 (30.53)	31 (38.75)	9 (17.65)	0.033
Discordant	19 (14.50)	13 (16.25)	6 (11.76)	
Concordant (+ / +)	53 (40.46)	26 (32.50)	27 (52.94)	
Missing	19 (14.50)	10 (12.50)	9 (17.65)	
Partnership HIV serostatus composition				
Concordant (-/-)	84 (64.12)	55 (68.75)	29 (56.86)	0.007
Discordant	7 (5.34)	1 (1.25)	6 (11.76)	
Concordant (+ / +)	3 (2.29)	0 (0.00)	3 (5.88)	
Missing	37 (28.24)	24 (30.00)	13 (25.49)	
Partnership HIV disclosure				
Both members disclosed	90 (68.70)	51 (63.75)	39 (76.47)	0.409
One member disclosed	17 (12.98)	11 (13.75)	6 (11.76)	
Neither member disclosed	21 (16.03)	16 (20.00)	5 (9.80)	
Missing	3 (2.29)	2 (2.50)	1 (1.96)	

### Demographic characteristics

Comparing the individual-level characteristics (Table 1 ) and partnership-level characteristics (Table 2) across both sites and separately for San Francisco and Montreal revealed several notable differences. The study population in Montreal was significantly older than that in San Francisco, with a median age of 37 (IQR = 16.0) compared to 26 (IQR = 5.7). Additionally, participants in Montreal reported a higher self-reported HCV prevalence (65% vs. 48%, *p* = 0.003) and self-reported HIV prevalence (11% vs. 4%, *p* < 0.001) compared to those in San Francisco.

These differences extended to partnership-level characteristics (Table 2). In Montreal, the partnership age composition skewed older, with 71% of partnerships involving individuals over the age of 30, in contrast to San Francisco, where 71% of partnerships involved individuals under 30 (*p* < 0.001). The partnership gender composition was similar across both sites, with most partnerships being male-male (53% in San Francisco, 63% in Montreal) and fewer being female-female partnerships (21% in San Francisco, 12% in Montreal).

Overall, most partnerships reported living together in the past month (66% in San Francisco and 59% in Montreal). In comparison, a smaller proportion reported having engaged in sexual activity with their partner during the same period (25% in San Francisco and 29% in Montreal).

### Injecting-related behaviors

Injecting-related behaviors were assessed at the individual level and are summarized in Table 1. Partnership-specific behaviors (e.g., number of days injected with a partner in the past month) and individual-specific behaviors (e.g., number of days injected in the past month without a partner) were examined.

Regarding sitewide differences, individuals in San Francisco reported injecting more frequently in the past month (median number of days 30 (IQR = 6.00) vs. 28 (IQR = 15.00), *p* = 0.017). Beyond the partner they participated in the study with, individuals in San Francisco reported having more injecting partners in the past month compared to those in Montreal (median number of other partners 4 (IQR = 9.00) vs. 1 (IQR = 2.00), *p* > 0.001). Both sites, however, were similar in terms of the number of days injected with their partner in the past month and the number of times per day injected with their partner in the past month.

### HCV testing and diagnosis

HCV status was self-reported, and participants were asked whether they disclosed their status to their injecting partner. Among participants who reported ever having an HCV test, 92 individuals (57%) self-reported a positive diagnosis based on having ever received a positive RNA test result, 61 individuals (38%) reported a negative diagnosis, and 9 individuals (6%) had a missing or unknown HCV infection status. The proportion of participants reporting positive HCV diagnoses was higher in Montreal compared to San Francisco (65% vs. 48%, *p* = 0.003).

Across both sites, there were 103 partnerships where both members disclosed their HCV status (79%), 16 partnerships where only one partner disclosed (12%), and 6 partnerships where neither partner disclosed (5%). Qualitatively, San Francisco had more one-way disclosures than Montreal (14% vs. 10%), while Montreal had more mutual disclosures than San Francisco (82% vs. 76%). Neither member disclosure behaviors similar across both sites. Mutual HCV disclosure occurred in 79% of partnerships, with higher rates in HCV-concordant positive partnerships (+ / +) (40%). Disclosure was lower in discordant partnerships (15%) and HCV-negative concordant partnerships (-/-) (30%).

**Fig. 1:**
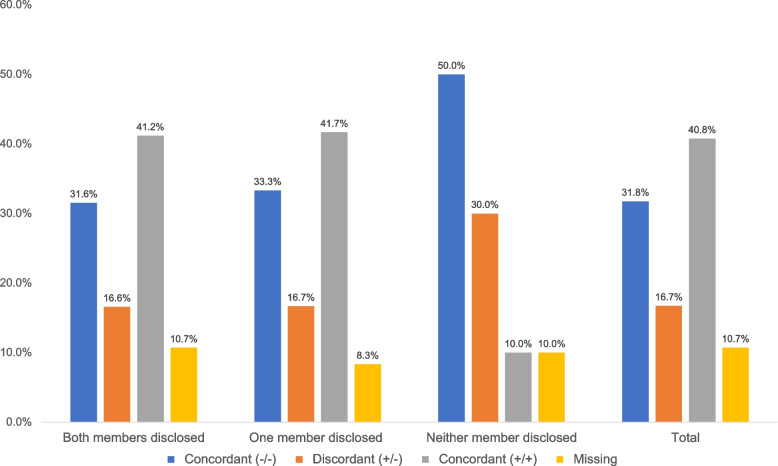
The figure displays the distribution of HCV status disclosure within injecting partnerships (N=131), categorized by self-reported partnership HCV serostatus: concordant negative (-/-), discordant (+/-), concordant positive (+/+), and missing. Disclosure is grouped into three categories: mutual disclosure (both partners disclosed their status), one-way disclosure (only one partner disclosed), and no disclosure (neither partner disclosed). Mutual disclosure was highest in concordant positive partnerships (41.7%) and lowest in discordant partnerships (16.7%). In contrast, no disclosure was most common in concordant negative partnerships (50.0%) and least common in concordant positive partnerships (10.7%). These findings highlight how disclosure behaviors vary by partnership HCV status, with higher mutual disclosure occurring when both partners self-report a positive HCV status

HCV disclosure by partnership self-report status illustrates the distribution of HCV status disclosure by self-reported HCV status (*p* = 0.61). Mutual disclosure was highest (41%) in concordant HCV-positive partnerships and lowest (17%) in discordant partnerships. One-way disclosure was also high (42%) among concordant HCV-positive partnerships and lower (17%) among discordant partnerships. Qualitatively, the disclosure results across partnership HCV status were similar between mutual and one-way disclosure (Fig. 1). Additionally, among concordant HCV negative partnerships, 50% reported that neither member disclosed, compared to 10% of concordant HCV positive partnerships reporting no disclosure.

In contrast, HIV status went undisclosed more frequently between partners than HCV status (14% vs. 4%), despite the majority of participants reporting knowledge of their HIV status. Most participants who reported an HIV test result indicated a negative diagnosis (78%), which was significantly higher than the negative HCV diagnosis rate (38%).

Finally, lifetime HCV treatment access among the San Francisco sample was low overall, with only eight individuals (3%) reporting access to HCV treatment. HCV treatment access was not measured in the Montreal sample. Discrepancies between perceived and actual HCV status were not assessed in this study but could play a role in risky behaviors. HIV disclosure rates were generally lower than HCV disclosure rates.

## Discussion

Our findings highlight the high rates of HCV disclosure within injecting partnerships, particularly in concordant positive partnerships. This suggests that injecting partners play a key role in encouraging safer injecting practices and may serve as a source of support for managing HCV. We observed high HCV testing and disclosure rates within partnerships involving PWID in San Francisco and Montreal. Across both sites, many partnerships witnessed mutual disclosure of HCV status, with similar patterns observed for one-way disclosures. Notably, concordant HCV-positive partnerships exhibited the highest rates of mutual disclosure, while discordant partnerships had the lowest. Additionally, the proportion of partnerships where neither member disclosed their HCV status was highest among concordant HCV negative partnerships, contrasting with the lowest occurrence among concordant HCV positive partnerships. These findings suggest that, despite a generally high prevalence of HCV disclosure, variations in disclosure behaviors become more evident when considering the HCV status of individuals within injecting partnerships.

Our findings align with existing literature on HCV-status disclosure among PWID in high-income urban settings. For instance, a study in New South Wales, Australia reported that approximately three-quarters of participants disclosed their HCV status to non-injecting partners or family members [22]. Similarly, within urban environments in Hungary and Lithuania, high rates of HCV-status disclosure were documented, with notable differences based on cultural contexts and ethnic groups [25]. These results emphasize the importance of investigating correlates of HCV disclosure within US urban contexts, including interpersonal factors and individual characteristics like race or ethnicity.

Increased HCV status disclosure has the potential to inform individuals' risk behaviors, facilitating safer injecting practices and reducing opportunities for HCV transmission [21, 26]. However, fewer studies have explored the factors associated with HCV disclosure compared to HIV disclosure and how these factors can inform strategies for enhancing HCV disclosure and preventing transmission. Comparing HCV-status disclosure to HIV-status disclosure, we found that overall HIV disclosure rates were low in both San Francisco and Montreal despite most participants knowing their HIV serostatus. This disparity may be attributed to the considerably higher population rates of HCV infection in this population compared to HIV; in 2019, the HCV seroprevalence among PWID in San Francisco was 67.4%, compared to a 10.1% seroprevalence of HIV [27, 28]. Similarly, in 2017 the HCV seroprevalence among PWID in Montreal was 69%, compared to a 21.5% seroprevalence of HIV in 2019 [29, 30].

However, a crucial point is the distinction between HCV antibody testing and viral load (RNA) testing [31]. Antibody tests indicate past exposure, while RNA tests confirm active infection, and encouraging disclosure based on antibody status could lead to unnecessary stigma, particularly for individuals who have cleared the infection [32]. The increasing availability of point-of-care RNA testing may help mitigate this issue and allow individuals to base disclosure decisions on more accurate indicators of their current infection status [33, 34]. While both HCV and HIV disclosure are influenced by stigma and can impact interpersonal relationships, the underlying factors affecting disclosure differ. HIV disclosure studies often focus on sexual partners, which may not be directly applicable to understanding HCV status disclosure due to the lower probability of sexual transmission of HCV [35, 36]. Moreover, legal mandates require HIV disclosure in specific healthcare settings, potentially affecting individuals' willingness to disclose their HCV status, whereas no such mandates exist for HCV [35, 37]. This difference impacts how stigma and trust influence disclosure decisions in injecting partnerships. Incorrect perceptions of HCV status, such as believing both partners are positive when one is negative, may increase transmission risk [38]. This underscores the need for education on the difference between antibody and RNA testing to promote safer behaviors.

Disclosure between partners can also provide an opportunity to harness existing discussions around HCV status within partnerships to develop harm reduction strategies. As seen in peer-supported prison screening programs, utilizing close relationships to promote HCV testing and care could be a key approach to improving outcomes for PWID [39]. In particular, disclosure events could serve as touchpoints for health promotion efforts, encouraging regular HCV testing and repeat testing, especially given the potential for spontaneous clearance or reinfection [40]. Centers for Disease Control and Prevention (CDC) guidelines recommend that PWID undergo HCV testing every six months, making these moments crucial for clarifying any misunderstandings about current infection status [41]. Regular RNA testing, in particular, can provide the most accurate reflection of current infection status, helping partners take informed steps to prevent reinfection and transmission [42]. This approach ensures that partners are not only aware of their current health status but also empowered to stay engaged in ongoing care.

The stigma associated with HCV disclosure should also be considered when interpreting our findings. Negative stereotypes among healthcare providers can stigmatize PWID, affecting the quality of healthcare they receive [43]. Discriminatory practices can deter individuals from engaging in medical care and exacerbate disengagement from the HCV diagnostic continuum [44, 45]. As other studies have demonstrated, reductions in HCV-related stigma and increases in self-care during HCV treatment may encourage more open disclosure of HCV status, which could further support engagement with care among PWID [46]. Addressing these psychological barriers in intervention strategies could improve treatment outcomes by fostering a supportive environment for HCV disclosure and self-management.

Successful interventions could aim to provide services without judgment and consider the experiences of PWID in healthcare settings [47]. Testing in community-based settings and harm reduction and prevention services present a promising avenue to increase disclosure rates and improve access to care [48]. Integrating strategies within medication-assisted treatment programs can further enhance support for individuals navigating the complexities of HCV disclosure and treatment, fostering a more inclusive and practical approach to healthcare delivery [49, 50].

Several limitations of our study should be acknowledged. First, the sample size was relatively small compared to some multi-site studies, which may limit the generalizability of the findings, especially in settings outside of San Francisco and Montreal. The urban contexts of San Francisco and Montreal, both of which have well-established harm reduction services and HCV testing infrastructure, may result in higher disclosure rates than would be observed in more rural or underserved areas. This may reflect the influence of local initiatives aimed at HCV reduction, which could have contributed to increased awareness and willingness to disclose HCV status among participants. Second, cohort effects occurring due to concerted local efforts for HCV reduction in both cities might have influenced the findings, as heightened awareness and easier access to testing could lead to higher rates of disclosure compared to other locations. Additionally, the cross-sectional design limits our ability to draw conclusions about causality or changes in disclosure behaviors over time. Third, limited data on lifetime HCV treatment access, which was only collected for the San Francisco sample, restricts our ability to generalize findings about treatment-related disclosure behaviors to both study sites. Including more detailed information on treatment histories, including the type of treatment received (e.g., direct-acting antivirals), could have provided valuable insights into how access to treatment influences disclosure decisions. Additionally, we did not assess whether participants were treated with DAAs, which could affect their current infection status. Including this information would provide a more accurate denominator for HCV disclosure rates. Finally, the use of self-reported HCV status may introduce bias, as there could be discrepancies between perceived and actual infection status. This limitation is particularly relevant given the misunderstanding that can occur between antibody and RNA test results, leading to potential misreporting. Future studies should incorporate both self-reported and serologically confirmed HCV status to address this potential source of bias and offer a more accurate picture of disclosure behaviors.

Our findings highlight an opportunity to harness existing discussions around HCV status within partnerships to develop harm reduction strategies. In particular, disclosure events could serve as touchpoints for health promotion efforts – to encourage regular HCV testing, HCV treatment, and harm reduction. also be leveraged as key moments to encourage regular HCV testing, particularly RNA testing, which provides the most accurate reflection of current infection status. This approach ensures that partners are not only aware of their current health status but can also take informed steps to prevent reinfection and transmission. Regular testing as part of a comprehensive harm reduction strategy could reduce misconceptions around serostatus and support both partners in staying engaged in ongoing care. Encouraging result sharing regardless of serostatus could bridge the gap among the 13% who disclosed their HCV status to a partner unaware of the result. This approach could involve injecting partners more actively in the HCV diagnosis and treatment continuum, facilitating support for individuals with positive diagnoses and prevention strategies for those with negative diagnoses. Leveraging the protective effects of HCV status disclosure, independent of the actual status, could enhance risk reduction efforts [21].

In conclusion, our study sheds light on HCV status disclosure within injecting partnerships among PWID in urban settings. Future steps could involve promoting HCV status disclosure independently of serostatus, offering partner HCV testing and disclosure counseling, and utilizing injecting partners as resources to enhance prevention and treatment strategies. Additionally, investigating the role of gender and racial dynamics in disclosure behaviors, as well as exploring how broader social networks beyond injecting partnerships influence these behaviors, could provide valuable insights for improving intervention approaches. These approaches can increase HCV testing frequency and reduce forward transmission in this vulnerable population.

## Conclusion

The high rates of HCV disclosure observed in this study highlight the importance of injecting partnerships in HCV care and prevention. However, improving awareness around the nuances of antibody and viral load testing is critical to ensure accurate disclosure and reduce the risk of transmission. Future interventions should aim to increase RNA testing access and address misperceptions around HCV status.

## Data Availability

The datasets used and/or analysed during the current study available from the corresponding author on reasonable request.
